# A Rare Case of Pseudo-Mirizzi Syndrome Presenting With Acute-on-Chronic Cholecystitis and Hepatic Abscesses

**DOI:** 10.7759/cureus.65031

**Published:** 2024-07-21

**Authors:** George G Kidess, Kenan Abou Chaer, Abdallah Almawazreh, Jarrett J Weinberger

**Affiliations:** 1 Department of Medicine, Wayne State University School of Medicine, Detroit, USA; 2 Internal Medicine, Wayne State University Detroit Medical Center, Detroit, USA; 3 Internal Medicine, Wayne State University/ Detroit Medical Center, Detroit, USA

**Keywords:** acute cholangitis, hepatic abscess, chronic cholecystitis, acalculous cholecystitis, mirizzi syndrome

## Abstract

Mirizzi syndrome (MS) is an uncommon cause of gallstone disease caused by calculous cholecystitis resulting in extrinsic obstruction of the common bile duct, causing concurrent obstructive jaundice. An acalculous variant of MS, at times referred to as pseudo-MS, occurs even more rarely. We present the case of a patient who was found to have pseudo-MS complicated by several hepatic microabscesses. The patient was managed with an endoscopic retrograde cholangiopancreatography (ERCP) with sphincterotomy and eventual cholecystectomy, with histopathology of the gallbladder confirming chronic cholecystitis. To our knowledge, the case presented here is the first in literature that identified pseudo-MS in a patient with pathology-confirmed chronic cholecystitis, and the first to be associated with hepatic abscesses; which usually occur with calculous rather than acalculous biliary disease.

## Introduction

Mirizzi Syndrome (MS) is a rare cause of symptomatic gallstone disease with an incidence of less than 1% annually, caused by acute or chronic calculous cholecystitis that eventually causes extrinsic compression and obstruction of the common hepatic duct or common bile duct, leading to simultaneous obstructive jaundice [1]. An acalculous variant of MS, in some papers referred to as pseudo-MS, occurs due to local inflammation of the gallbladder that obstructs the cystic duct in the absence of a gallstone and has been only described in a handful of cases in the literature [2,3].

We present a novel case of acute-on-chronic acalculous cholecystitis leading to pseudo-MS, complicated by the presence of hepatic abscesses.

## Case presentation

The patient is a woman in her 60s with a past medical history of gastroesophageal reflux disease (GERD) who presented to the emergency department with 3 days of epigastric, right upper abdominal quadrant (RUQ), and right flank pain. The pain was intermittent in nature, which she attributed to having gas, and was rated 7 out of 10. She endorsed that she previously experienced this pain, which was relieved with omeprazole, however, that did not suffice during this episode which prompted her to seek medical assistance. In the emergency department, she was hemodynamically stable, and not in distress, and physical examination was significant for RUQ tenderness with a positive Murphy’s sign. The rest of the examination was unremarkable, with no peritoneal signs, organomegaly, costovertebral angle tenderness, or signs of scleral icterus. Labs showed leukocytosis of 19.5 with an elevated absolute neutrophil count of 15.1. Hyperbilirubinemia was also noted with a total bilirubin of 1.65 and direct bilirubin of 0.30.

**Figure 1:**
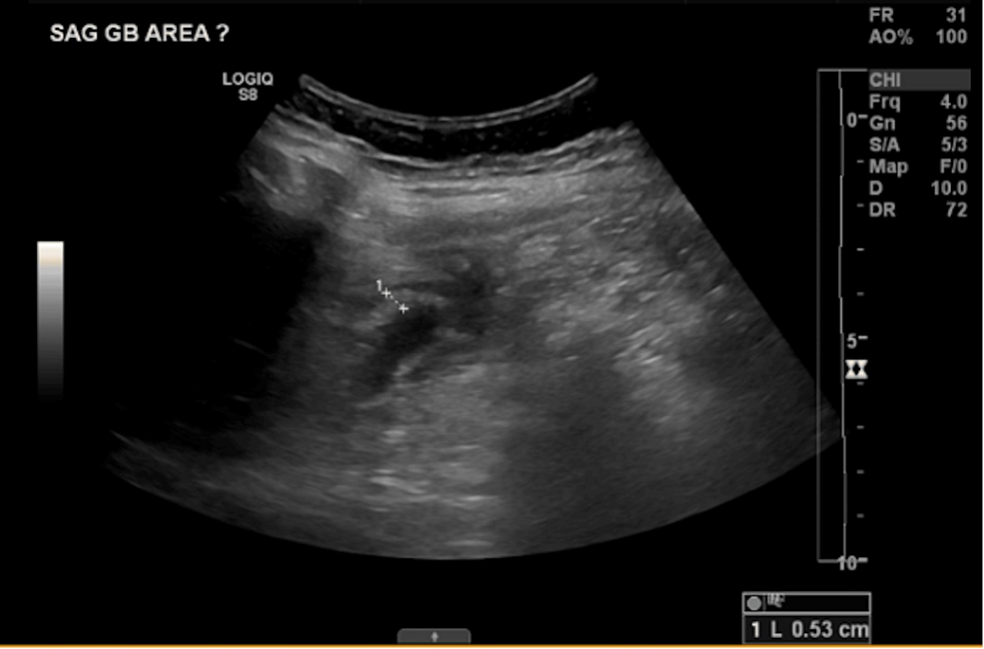
Abdominal ultrasound showing thickening of gallbladder wall of 53 mm

**Figure 2:**
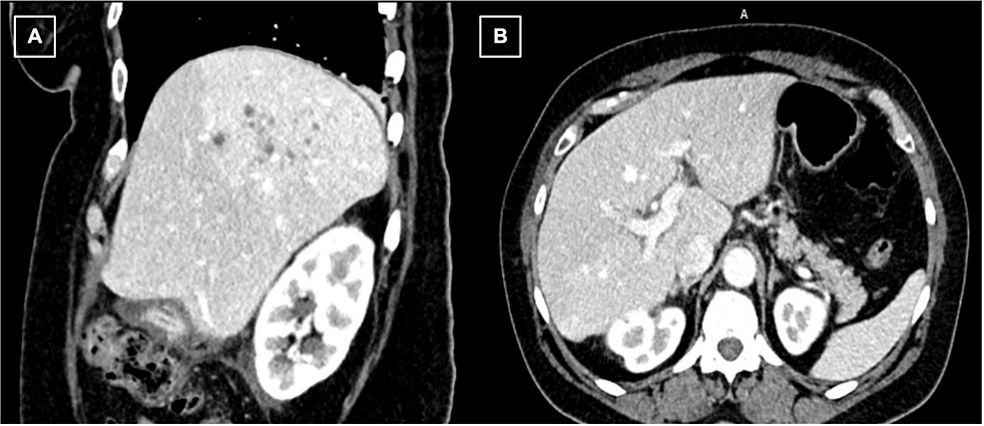
Sagittal (A) and axial (B) CT abdomen and pelvis showing edematous gallbladder, hepatic abscesses, and dilated intrahepatic bile ducts

An abdominal ultrasound (Figure 1) was obtained and showed extensive gallbladder wall thickening with echogenic foci in the lumen representing acute cholecystitis. CT abdomen and pelvis (Figure 2) on admission was also done confirming acute cholecystitis, in addition to findings of intrahepatic biliary duct dilatation in the right hepatic lobe and multiple fluid collections concerning hepatic abscesses. These findings were concerning for acute cholangitis complicated with multiple hepatic abscesses and led to the patient being admitted with gastroenterology and infectious diseases consultations.

**Figure 3:**
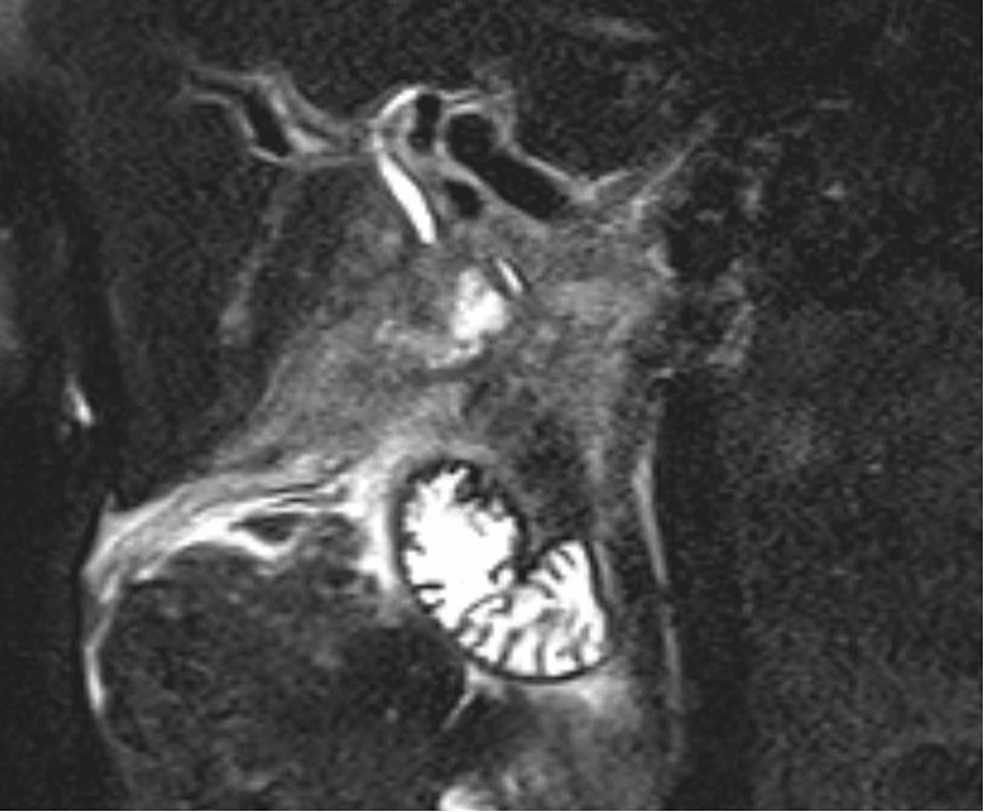
: Magnetic retrograde cholangiopancreatography (MRCP) showing extrinsic compression of the mid-common bile duct (CBD) by the inflamed gallbladder

**Figure 4:**
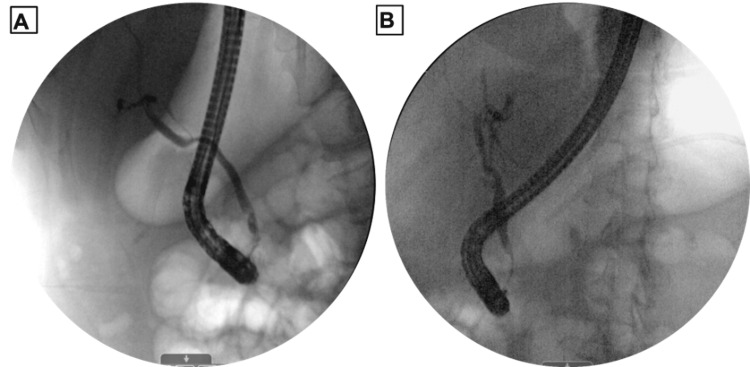
A) Preoperative endoscopic retrograde Cholangiopancreatography (ERCP) showing external compression of the mid-distal common bile duct (CBD). B) Postoperative ERCP showing resolution of CBD compression

Upon admission, magnetic retrograde cholangiopancreatography (MRCP, Figure 3) was done and showed signs of extrinsic compression of the common bile duct (CBD) by the inflamed gallbladder resulting in a focal stricture that was concerning for acute cholangitis. Based on these results, endoscopic retrograde cholangiopancreatography (ERCP, Figure 4) was completed the following day which also showed external compression of the CBD due to acute cholecystitis resembling MS, and biliary sphincterotomy was performed with biliary stent placement.

**Figure 5:**
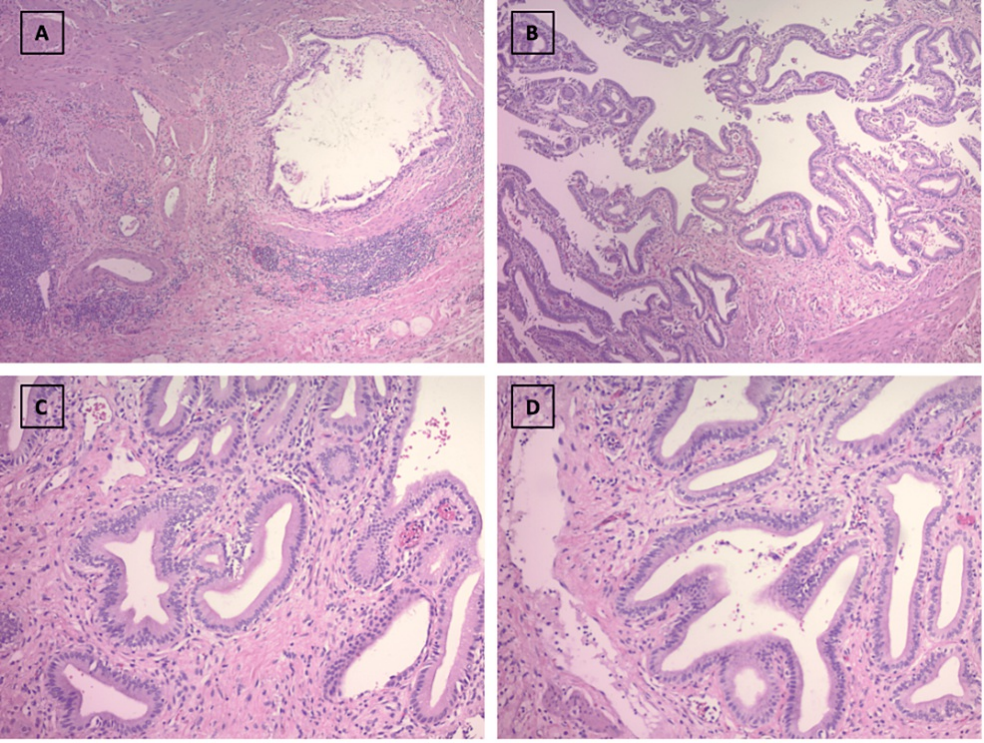
Hematoxylin and eosin (H&E) staining Staining in low (A and B) and high (C and D) magnification of the gallbladder showing infiltration with monocytes, edema, and diverticula consistent with chronic cholecystitis.

Given the findings of acute cholecystitis, two days after the ERCP the patient underwent a subtotal cholecystectomy. Intraoperatively, the gallbladder was found to be significantly fibrous and woody consistent with acute-on-chronic cholecystitis. This was confirmed by pathology of the gallbladder which showed chronic cholecystitis as well as edema and diverticula (Figure 5). Postoperatively, the patient did well and was discharged on intravenous ceftriaxone and oral metronidazole for 6 weeks per infectious diseases consultation to appropriately treat the hepatic abscesses.

**Figure 6:**
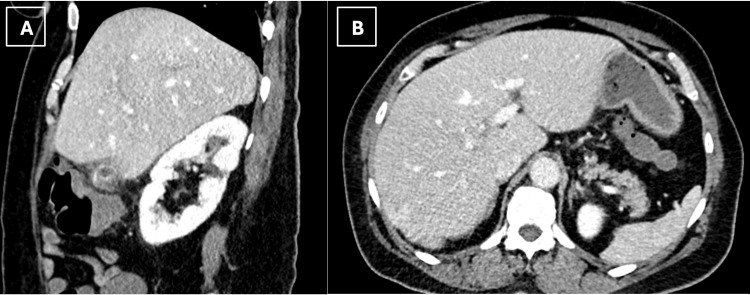
Sagittal (A) and axial (B) CT abdomen and pelvis showing recurrent cholecystitis after subtotal cholecystectomy and interval resolution of previous hepatic abscesses

Unfortunately, one week after discharge, the patient returned to the emergency department with recurrent RUQ abdominal pain, nausea, and vomiting, and was found to have elevated transaminases and alkaline phosphatase. She was evaluated by general surgery and a decision was made to perform a remnant cholecystectomy, which was completed with remarkable difficulty due to intensive scarring and inflammation. During the same admission, a CT scan of the abdomen and pelvis (Figure 6) showed interval resolution of the hepatic microabscesses after two weeks of antibiotic coverage. The patient did well postoperatively and was discharged on the same antibiotic course to complete the treatment of the abscesses with close outpatient follow-up with infectious diseases.

## Discussion

Since pseudo-MS is an incredibly rare phenomenon, its risk factors, presentation, and consensus on treatment have yet to be defined. In one retrospective study, RUQ and epigastric pain, hyperbilirubinemia, and leukocytosis were present in all patients such as in our case [4]. Although jaundice is a classic clinical sign of biliary obstruction, it was not observed in our patient. This is consistent with the cited study showing that only twenty percent of the patients presented with jaundice, which it concluded was either due to incomplete biliary obstruction or the fact that jaundice might present later in the disease course [4]. Identification of pseudo-MS by imaging has reportedly been challenging, especially with MRCP which has only successfully identified the condition in a few cases, including this one and one small retrospective study [2,4-6].

Notably, all reported cases of pseudo-MS at the time of writing this report have been identified in patients with acute cholecystitis [7,8], and to our knowledge, this case is the first reported in the literature of pathology-confirmed chronic cholecystitis. The chronic inflammation might have been the underlying reason for a subtotal cholecystectomy not sufficing in this patient, requiring a complete cholecystectomy for the resolution of her symptoms. Additionally, our case is the first reported that also involves hepatic abscesses, which are commonly caused by calculous biliary disease and only have been reported in acalculous cholecystitis secondary to gallbladder perforation [9,10].

Although no specific management and treatment guidelines have been proposed given the scarcity of data on pseudo-MS, the majority of cases are managed in a manner similar to acute cholangitis especially since they present similarly. The majority of patients in literature with pseudo-MS are treated surgically with a cholecystectomy, while a small amount underwent a percutaneous cholecystostomy to relieve the obstructive jaundice [2,4]. This is similar to our case in which stent placement via ERCP was attempted prior to the cholecystectomy. In one case conservative management with IV fluids and antibiotics was sufficient for gradual patient improvement and was successful even at follow-up months later [6].

## Conclusions

Pseudo-MS can be challenging to diagnose given the current scarcity of research and diagnostic guidelines, which makes the identification of specific risk factors, symptoms, and management options more difficult. We hope that the case presented may shed light on this unusual phenomenon and its potential implications, highlight novel findings for this disease, and encourage more research.
